# A Rare Association of Autoimmune Hemolytic Anemia with Gastric Adenocarcinoma

**DOI:** 10.1155/2017/8414602

**Published:** 2017-10-09

**Authors:** Kavita Agrawal, Flores Alfonso

**Affiliations:** ^1^Department of Internal Medicine, Overlook Medical Center, Summit, NJ 07901, USA; ^2^Department of Pathology, Overlook Medical Center, Summit, NJ 07901, USA

## Abstract

An 80-year-old male presented with dyspnea on exertion for at least two months. He also complained of progressive dysphagia and weight loss of 35 pounds over the last eight months. Initial blood tests showed hemoglobin of 6.1 g/dl, reticulocytes count of 19.7%, total bilirubin of 3.2 mg/dl, lactate dehydrogenase of 600 U/L, and haptoglobin of less than 8 mg/dl, and direct Coombs test was positive for warm immunoglobulin G. The impression was autoimmune hemolytic anemia (AIHA). The evaluation of dysphagia with esophagogastroduodenoscopy revealed a single irregular 4 cm malignant appearing ulcerated mass at the incisura angularis of the stomach. The mass was confirmed as adenocarcinoma on biopsy. Diagnostic laparoscopy was positive for malignant cells and he was diagnosed with stage IV adenocarcinoma of the stomach. Other extensive workup to determine the etiology of AIHA was negative (described in detail below). Surgery was deferred primarily due to metastasis of cancer. Initially, hemoglobin was stabilized by intravenous methylprednisolone, high dose immunoglobulins, and packed red blood cell transfusions. After a few weeks, hemoglobin started trending down again. The patient was weaned off steroids and paradoxically IgG-mediated autohemolysis was controlled with the initiation of palliative chemotherapy. Our case highlights a rare occurrence of AIHA in association with gastric adenocarcinoma.

## 1. Case Report

An 80-year-old African American male presented with an insidious onset of dyspnea on exertion for at least two months with progressive worsening over two to three weeks. It was also associated with orthopnea and lower extremity swelling. Prior to this presentation, he used to walk one block or one flight of stairs without getting short of breath. Presently, however, he had difficulty walking even 30 feet on level ground or climbing few steps of a stair.

He also complained of difficulty swallowing for eight months. Initially noticed with solid foods, it had progressed such that, now, even liquids had to be swallowed slowly. He noted that he was unable to swallow pills; this made him feel like a pill is stuck in the middle of the chest and so he stopped taking his medications. He also reported a 35-pound weight loss over the last eight months. He denied odynophagia, nausea, vomiting, constipation, or abdominal pain. He denied rash, arthralgias, photosensitivity, dry eyes, dry mouth, joint swelling, or family history of an autoimmune or rheumatologic disease.

He had past medical history of hypertension. He denied a prior history of anemia or blood transfusions. He had no past surgical history. He never had an upper endoscopy or colonoscopy. He had no known allergies. His only medication was amlodipine, which he stopped taking eight months earlier due to dysphagia. He had a smoking history of 5 pack-years but had stopped smoking 30 years ago, he had occasional alcohol use of 1-2 glasses of wine during weekends, and he denied illicit drugs use. He had no significant family history. He had not seen his primary care doctor in at least a year. He lived alone at home and was independent in activities of his daily living.

Physical examination revealed a thin cachectic male with no apparent distress. His pulse was 76 beats per minute, blood pressure 159/80 mmHg, respiratory rate 19 breaths per minute, and oxygen saturation 100% on two-liter nasal cannula. His body mass index was 19.9 kg/m^2^. Pale conjunctiva and icteric sclera were noted. There was no lymphadenopathy. Minimal bibasilar crackles were auscultated on lung exam. Heart sounds were normal and rhythm was regular. No murmurs were heard. The abdomen was soft, nontender, and nondistended with no hepatosplenomegaly. On bilateral lower extremities, 1+ pitting ankle edema was present. No rash or joint swelling was present.

Investigations (refer to Table 1) revealed a hemoglobin level of 6.1 g/dl which dropped to 5.1 g/dl in the next 12 hours with no fluids, white blood cell count of 6160/*μ*l, platelet count of 348 × 10^3^/*μ*l, mean corpuscular volume of 113.8 fl, and a reticulocyte count of 19.1%. Peripheral smear showed moderate red cell anisopoikilocytosis and polychromasia. Neutrophils, lymphocytes, and platelets were morphologically normal.

Further workup showed total bilirubin of 3.2 mg/dl, conjugated bilirubin of 1.2 mg/dl, serum lactate dehydrogenase of 600 U/L, and haptoglobin of less than 8 mg/dl, and direct Coombs test was positive for warm immunoglobulin G. Serum protein electrophoresis with immunofixation revealed no abnormal monoclonal protein. Rapid human immunodeficiency virus test was negative. Anti-nuclear antibodies were positive, but other antibodies as shown in Table 1 were all negative. As mentioned above, there was no history or exam findings suggestive of rheumatologic diseases. Therefore, the positive ANA titer was considered incidental.* Mycoplasma* antibodies were also negative. Other test results are shown in Table 1. A diagnosis of warm IgG-mediated autoimmune hemolytic anemia (AIHA) was made.

On day two of hospitalization, further tests were done to rule out underlying lymphoproliferative disorders likely contributing to AIHA. Computed tomography of the abdomen and pelvis with oral and intravenous contrast showed no frank evidence of lymphoproliferative disease. Computed tomography of the chest with intravenous contrast revealed an anterior mediastinal soft tissue mass with dystrophic calcifications, bilateral pleural effusions, and mediastinal lymphadenopathy. Considerations for soft tissue mass included thymic neoplasm and lymphadenopathy.

Bronchoscopy was performed on day six of hospitalization. Endobronchial ultrasound was used to perform biopsy of the anterior mediastinal soft tissue mass and subcarinal and mediastinal lymph nodes. Histological review showed cells consistent with lymph node sampling but no malignancy was identified. Flow cytometry from the biopsy showed no evidence of malignancy.

Dysphagia was worked up on day eight of hospitalization with an esophagogastroduodenoscopy. At 25 cm from the incisors, a tight benign appearing esophageal stricture causing severe obstruction was encountered. This was dilated using 8, 10, and 11 mm balloon dilators. The obstruction was then able to be traversed by scope. At the incisura angularis of the stomach, a single 4 cm mass was encountered. It had irregular margins and an ulcerated surface and did not have any bleeding. The gastroesophageal junction, pylorus, duodenal bulb, and second part of the duodenum appeared to be normal. A biopsy from an ulcerated mass was taken.

**Figure 1:**
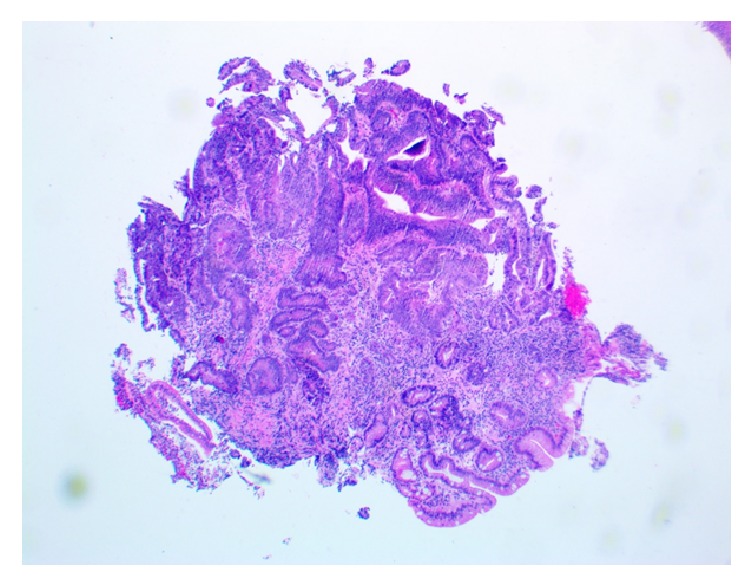
Stomach incisural mass biopsy on low microscopic power showing gastric mucosa with intestinal metaplasia and involvement by adenocarcinoma with invasion into the submucosa.

**Figure 2:**
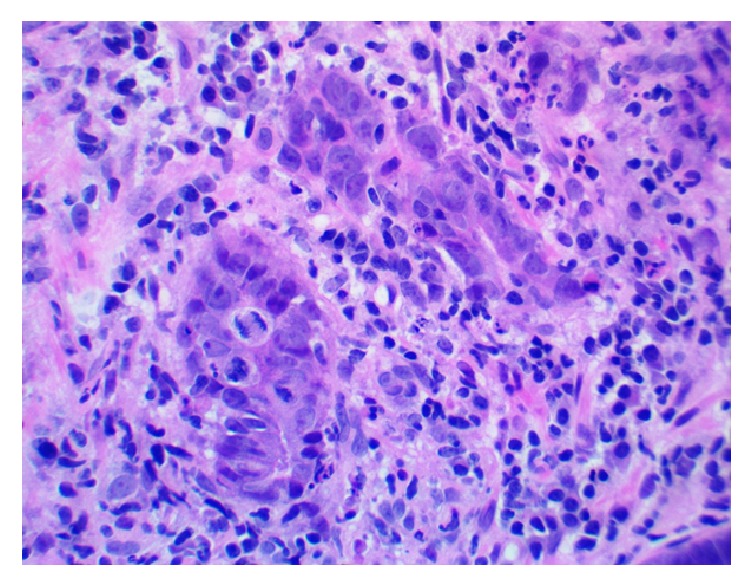
Stomach incisural mass biopsy on high microscopic power showing disorganized groups of pleomorphic cells with nuclear atypia, large nucleoli, and mitotic activity.

Pathological review of the incisural mass biopsy revealed nuclear atypia, mitotic activity, and mucinous cells in clusters without clear epithelial formations invading the submucosa (Figures 1 and 2). A diagnosis of invasive adenocarcinoma was made. The adenocarcinoma of the stomach was further staged in the third week after the initial presentation. A diagnostic laparoscopy with peritoneal washings was performed. Cytology from the washings was positive for malignant cells consistent with metastatic adenocarcinoma. A diagnosis of stage IV adenocarcinoma of the stomach was made. Given the findings from the laparoscopy, the carcinoma was felt to be nonresectable.

## 2. Treatment and Patient Course

On the day of presentation, after the diagnosis of AIHA was made, he was started on intravenous methylprednisolone 1 mg/kg body weight daily for five days and was transfused with cross-matched compatible packed red blood cells. Initially, his hemoglobin improved from 5.1 g/dl to 8.6 g/dl with the above regimen, but on day five of hospitalization, his hemoglobin gradually started declining even on steroids. By day seven, his hemoglobin had dropped to 6.6 g/dl. Therefore, he was started on high dose intravenous immunoglobulin 1000 mg/kg body weight for two days and his course of intravenous methylprednisolone 1 mg/kg body weight was extended for a total of ten days. He received a total of six units of packed red blood cells over a ten-day course of hospitalization. He was also given three doses of intravenous iron sucrose secondary to iron deficiency.

By day ten after the initial presentation, his hemoglobin level had stabilized at around 8 g/dl. His dyspnea had resolved. Also, his dysphagia had improved after the esophageal stricture dilatation. He was thus discharged from the hospital. At discharge, he was prescribed 50 mg of prednisone daily. The plan was to taper the daily dose of prednisone every week by 10 mg. The patient was asked to follow up with the oncologist.

On day 21 after the initial presentation, he was seen in the oncologist's office. At that time, his hemoglobin was 8.5 g/dl on the above steroid taper. With regard to his adenocarcinoma, the plan was to give him palliative chemotherapy regimen consisting of folinic acid, 5- fluorouracil, and oxaliplatin (FOLFOX) to be repeated biweekly for a total of six cycles.

On day 48 after the initial presentation, he received his first cycle of chemotherapy. At the time, he was being maintained on 15 mg oral prednisone daily. As of the writing of this report, he has received five of his six cycles of chemotherapy. His steroid requirement has dropped to 2.5 mg daily and hemoglobin has improved to around 10 g/dl. Initially, after the first chemotherapy cycle, LDH increased from 386 U/L to 612 U/L. However, from the second cycle onwards, LDH consistently trended down from 612 U/L to 369 U/L.

## 3. Discussion

AIHA is a clinical entity characterized by destruction of red blood cells due to the production of autoantibodies. It can be classified either as primary (idiopathic) or as secondary (when it is associated with another underlying condition). Lymphoproliferative disorders account for about half of the secondary causes of AIHA [1]. This includes non-Hodgkin's lymphoma and an estimated 10% of chronic lymphocytic leukemia (CLL) [2]. In CLL patients treated with purine analogs like fludarabine [3] and pentostatin [4, 5], the incidence of AIHA is even higher. Autoimmune diseases like systemic lupus erythematosus, rheumatoid arthritis, scleroderma, ulcerative colitis, and Crohn's disease are also a significant cause of AIHA [1]. Other causes include viral infections, blood transfusions, hematopoietic or solid organ transplantation,* Mycoplasma pneumoniae*, and various drugs like methyldopa, penicillins, cephalosporins, and NSAIDs [6]. AIHA's association with solid malignancies is rare [7]. In a 2010 meta-analysis, Joe et al. evaluated cases of AIHA associated with solid malignancies reported from the year 1945 to 2009. This included 52 case reports with ages ranging from 38 to 76 years old. The three most commonly reported cancers were renal cell carcinoma, Kaposi sarcoma (pre-HAART era), and non-small-cell lung cancers in that sequence [8]. AIHA has also been associated with a variety of other cancers including ovary, liver, colorectal, breast, stomach, uterus, prostate, and testis cancer [8].

When evaluating the causes of AIHA in our patient, we first focused on more commonly associated disorders. However, our extensive workup (as detailed above) did not reveal any evidence of lymphoproliferative disorder. Furthermore, autoimmune diseases were reasonably ruled out. The patient did not have any evidence of recent or active infection. He was not on any medications classically associated with AIHA. This raised our concern for rarer causes such as occult solid malignancy. Esophagoduodenoscopy was performed for the evaluation of dysphagia. This led to an incidental discovery of the gastric carcinoma.

There are some important features that suggest that gastric adenocarcinoma was involved in the pathogenesis of AIHA in our case. (1) The gastric carcinoma seems to have temporally preceded the development of AIHA. He initially noted weight loss eight months prior to presentation. This was presumably due to reduced oral intake due to dysphagia and adenocarcinoma itself. The symptoms such as shortness of breath that may be attributed to anemia were less than two months old. (2) Of the various therapies given in this patient for AIHA, the most hematological improvement was paradoxically observed after he received chemotherapy. The mechanisms underlying AIHA secondary to malignancies are not well known. It has been postulated that antibodies generated against tumor cells can cross-react with red blood cell antigens causing hemolysis [8].

Steroids are the mainstay of treatment for AIHA in addition to treating an underlying cause. Steroids are effective in 70–80% of patients [9–11]. The remaining 20–30% of steroid-unresponsive patients will require other treatment options [9, 10]. These include rituximab, splenectomy, immunosuppressive agents (like azathioprine, cyclophosphamide, and mycophenolate), intravenous immunoglobulins, and danazol [9]. As a last resort, high dose cyclophosphamide or alemtuzumab can be considered [9]. The data on how to treat AIHA when associated with an underlying solid tumor is sparse. In our literature search, we came across four case reports of AIHA secondary to gastric carcinoma [12–15]. In one case, steroids were intolerable due to underlying diabetes and the patient had refused surgical resection of cancer [12]. A rapid decrease in reticulocyte count and LDH was seen after the initiation of second-line therapy with rituximab [12]. In two other cases, patients were initially treated with steroids and hematological improvement was observed [13, 14]. However, either corticosteroid dependence or relapse of hemolysis was observed with time. Patients underwent surgical resection of cancer with or without splenectomy and thereafter remained in remission [13, 14]. In the last case, hemoglobin improved with steroids and adjunctive treatment with high dose intravenous immunoglobulins [15]. In our patient, initially, a similar observation was noted with steroids and intravenous immunoglobulins. However, after few weeks, hemoglobin started trending down. Hemoglobin improved and steroids were gradually weaned with the initiation of FOLFOX chemotherapy. This could possibly be due to reduction in the cancer antigenic load with the introduction of chemotherapy. AIHA secondary to solid tumors is thought to be less responsive to steroids and the treatment of the underlying condition is very important [7]. Based on the patient's response in our case, we believe that FOLFOX chemotherapy can be utilized as a potential treatment option to control autohemolysis associated with underlying gastric cancer.

## 4. Conclusion

Our report highlights a case of an 80-year-old male diagnosed with IgG-mediated autohemolysis in association with a metastatic gastric adenocarcinoma. The association of AIHA with solid tumors is rare. Also, this case adds to our limited literature on the treatment of AIHA secondary to gastric cancer. In our patient, there was a relapse of autohemolysis after an initial treatment with steroids and immunoglobulins. Hemoglobin was stabilized and steroids were successfully weaned with the initiation of FOLFOX chemotherapy. We believe that FOLFOX can be utilized as an alternative treatment for steroid-resistant AIHA with underlying gastric cancer.

## Figures and Tables

**Table 1:** Laboratory data.

	On admission	48 hours after admission	Reference range
White cell count (per *µ*l)	6160	8810	4500–11000
Hemoglobin (g/dl)	6.1	8.6	14.0–17.0 (men)
Hematocrit (%)	18.2	24.4	39.0–50.0
Red cell count (per pl)	1.60	2.46	4.20–5.70
Mean corpuscular volume (fl)	113.8	99.2	80.2–99.4
Platelet count (per *µ*l)	363,000	380,000	150,000–450,000
Differential count (%)			
Neutrophils	66.7		40.0–80.0
Lymphocytes	27.9		15.0–40.0
Monocytes	4.9		4.0–12.0
Basophils	0.2		0.0–2.0
Eosinophils	0.0		0.10–0.20
Sodium (mmol/l)	137	135	135–145
Potassium (mmol/l)	3.5	3.8	3.2–4.9
Chloride (mmol/l)	103	99	95–110
Carbon dioxide (mmol/l)	24	29	21–32
Glucose (mg/dl)	100	113	70–100
Blood urea nitrogen (mg/dl)	14	18	7–18
Creatinine (mg/dl)	1.3	1.1	0.6–1.3
Calcium (mg/dl)	7.8	8.0	8.5–10.2
Total protein (g/dl)	6.9	6.6	6.0–8.2
Albumin (g/dl)	2.7	2.6	3.4–5.0
Aspartate aminotransferase (U/liter)	25	21	15–37
Alanine aminotransferase (U/liter)	10	10	12–78
Alkaline phosphatase (U/liter)	80	68	45–117
Total bilirubin (U/liter)	3.4	2.7	0.0–1.1
Conjugated bilirubin (U/liter)	1.2		0.0–0.2
Lactate dehydrogenase (U/liter)	614	454	85–240
Haptoglobin (mg/dl)	<8		30–200
Reticulocyte count (%)	19.7		0.4–2.7
Prothrombin time (seconds)	15.5		12.0–15.0
International normalized ratio (seconds)	1.23		
Activated prothrombin thromboplastin time (seconds)	29.8		23.0–37.0
Folic acid (ng/ml)	4.8		4.0–18.0
Vitamin B12 (ng/l)	669		193–986
Iron level (*µ*g/dl)	56		65–175
Transferrin (mg/dl)	191		200–360
Saturation (%)	20		20–44
Ferritin (*µ*g/l)	93		5–244
IgG, *Mycoplasma pneumoniae*	0.70		≤0.90
IgM, *Mycoplasma pneumoniae*	0.36		≤0.90
Carcinoembryonic antigen (*µ*g/l)	3.3		0.0–3.0
CA 19-9 (U/ml)	4.6		0.0–35.0
Anti-nuclear antibody	Positive		Negative
Anti-double-stranded antibody	Negative		Negative
Ribonucleoprotein antibody	Negative		Negative
Anti-SS-A antibody	Negative		Negative
Anti-SS-B antibody	Negative		Negative
Anti-Jo-1 antibody	Negative		Negative
Anti-scleroderma-70 antibody	Negative		Negative
Anti-histone antibody	Negative		Negative
Anti-centromere antibody	Negative		Negative
Anti-Smith antibody	Negative		Negative
Myeloperoxidase antibody	Negative		Negative
Serine protease-3 antibody	Negative		Negative
